# Bacterial community of sediments under the Eastern Boundary Current System shows high microdiversity and a latitudinal spatial pattern

**DOI:** 10.3389/fmicb.2022.1016418

**Published:** 2022-09-30

**Authors:** Alexis Fonseca, Carola Espinoza, Lars Peter Nielsen, Ian P. G. Marshall, Victor A. Gallardo

**Affiliations:** ^1^Center for Electromicrobiology, Department of Biology, Aarhus University, Aarhus, Denmark; ^2^Department of Oceanography, University of Concepción, Concepción, Chile

**Keywords:** sulfur bacteria, sulfuretum, OMZ, anoxic sediments, microdiversity, bacterial community, *Desulfobulbaceae*, amplicon sequencing

## Abstract

The sediments under the Oxygen Minimum Zone of the Eastern Boundary Current System (EBCS) along Central-South Peru and North-Central Chile, known as Humboldt Sulfuretum (HS), is an organic-matter-rich benthic habitat, where bacteria process a variety of sulfur compounds under low dissolved-oxygen concentrations, and high sulfide and nitrate levels. This study addressed the structure, diversity and spatial distribution patterns of the HS bacterial community along Northern and South-Central Chile using 16S rRNA gene amplicon sequencing. The results show that during the field study period, the community was dominated by sulfur-associated bacteria. Indeed, the most abundant phylum was *Desulfobacterota*, while Sva0081 sedimentary group, of the family *Desulfosarcinaceae* (the most abundant family), which includes sulfate-reducer and H_2_ scavenger bacteria, was the most abundant genus. Furthermore, a spatial pattern was unveiled along the study area to which the family *Desulfobulbaceae* contributed the most to the spatial variance, which encompasses 42 uncharacterized amplicon sequence variants (ASVs), three assigned to *Ca*. Electrothrix and two to *Desulfobulbus*. Moreover, a very high microdiversity was found, since only 3.7% of the ASVs were shared among localities, reflecting a highly diverse and mature community.

## Introduction

In the Eastern Boundary Current System (EBCS) off the coasts of Chile and Peru, one of the world’s largest subsurface sulfidic benthic system occurs: the “Humboldt Sulfuretum”’ (HS) (Gallardo et al., 2016). While likely quite widespread in primeval oceans, *ca*. 3.5 Ga ago (Van Kranendonk et al., 2003; Ueno et al., 2008; Wacey et al., 2011; Schopf et al., 2017), these systems are rare and of limited extent in today’s Earth oceans. However, notwithstanding their reduced extension, *ca*. 1.15 million km^2^ (Helly and Levin, 2004), their contribution to the productivity of the world’s oceans is disproportionately high. In this thus rather rare, nonetheless significant biome, benthic bacteria process a variety of sulfur compounds under the absence or very low levels of dissolved oxygen, but rather high levels of reduced sulfur compounds, nitrate, and phosphate (Fossing et al., 1995; Høgslund et al., 2008; Gallardo et al., 2013a).

In similar settings, i.e., sulfide-rich/oxygen-deficient seafloor and subsea sediments, bacterial communities are characterized by anaerobic bacteria, such as sulfate-reducing anaerobic forms and fermenters of *Chloroflexi*, *Proteobacteria*, *Firmicutes*, or *Candidatus* Atribacteria as well as methanogenic, and methanotrophic archaea (Orsi, 2018). These communities are thus well-differentiated from those living in oxic sediments (Zinger et al., 2011; Bienhold et al., 2016). Under sulfidic conditions, the availability of electron donors and acceptors, geochemical and sedimentological properties control the structure of bacterial communities (Fry et al., 2008; Hoshino et al., 2020). Furthermore, lithotrophic, and mostly autotrophic, sulfur-oxidizing bacteria (SOB) are conspicuous inhabitants of these environments, i.e., the “megabacteria” *Cand*. Marithioploca araucae (Salman et al., 2011), and the “macrobacteria” *Cand*. Venteria Ishoeyi (Fonseca et al., 2017), which generate extensive mats in the HS (Gallardo, 1977; Maier and Gallardo, 1984; Schulz et al., 1996; Ferdelman et al., 1997).

Previous work in the HS, using 16S rRNA amplicon sequencing, focused on the diversity of the bacterial community in selected areas of this system. Thereby, following a combination of the principles that sustain the Thienemann-Sanders time-stability hypothesis, Gallardo et al. (2016) suggested that the HS sublitoral bacterial community inhabiting the Central-South distribution area (∼36°s), corresponds to an evolutionarily mature, “biologically accommodated” community, a condition that is characterized by the lack of statistically dominant species (OTUs). Moreover, recent work in the Northern Mejillones Bay, located in the permanent mid-water oxygen-deficient upwelling system off Northern Chile, reports that members of *Anaerolineaceae*, *Thiotrichaceae*, *Desulfobulbaceae*, *Desulfarculaceae*, and *Bacteroidales* play an important role in the differentiation of sampling points in this area (Zárate et al., 2021).

The present work is a contribution to the knowledge on the structure and diversity of the HS bacterial community through sediment samples obtained off the coast of Chile from five localities distributed between *ca*. −20 and −36.10′, using 16S rRNA gene amplicon sequencing.

## Materials and methods

### Sediment core samples

**FIGURE 1:**
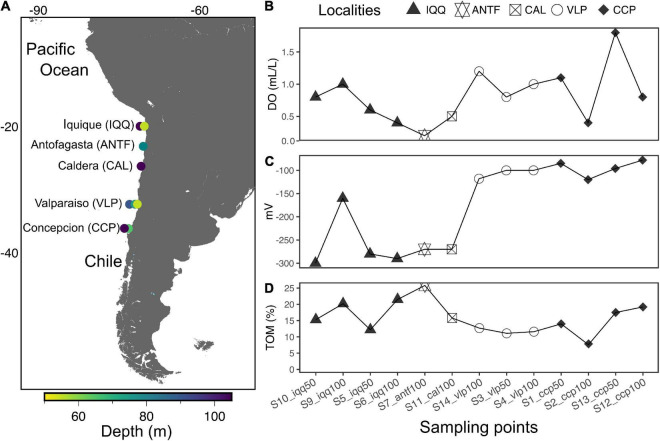
**(A)** Map of sampling points. **(B)** Dissolved oxygen (DO) of bottom water in ml/L. **(C)** Average redox potential of the first 5 cm of sediment, measured in millivolts (mV). **(D)** Average total organic matter (TOM) in percentage of the first 5 cm of sediment.

Off the coast of Chile sediment core samples were collected at approximately 50 and 100 m depth at five latitudes from −20 to −36 (Table 1), namely off Iquique (IQQ), Antofagasta (ANTF), Caldera (CAL), Valparaíso (VLP), and Concepcion (CCP) (Figure 1A). During samplings, the Northern IQQ, CAL, ANTF, and VLP substrates were evidently reduced and devoid of macrofauna, while at the Southern CCP station the scattered presence of polychetes and bivalves was noticeable.

**TABLE 1:** Metadata for the sampling points included in the study.

Season	Samples	Date*	Lat (S-)	Long (W-)	Depth (m)	Temp. °C	DO ml/L	Sediment** redox	Water redox	TOM%
Fall	S10_iqq50	14/05/2012	20°10,933	70°09,313	72	14	0.81	−300	−110	15.3
Fall	S9_iqq100	15/05/2012	20°10,531	70°10,964	97	13	0.96	−160	−8	20.2
Summer	S5_iqq50	13/01/2012	20°10,933	70°09,313	50	14.5	0.63	−280	−120	12.2
Summer	S6_iqq100	14/01/2012	20°10,531	70°10,964	100	14.1	0.4	−290	−135	21.5
Summer	S7_antf100	19/01/2012	23°37,489	70°25,481	100	16	0.16	−270	−77	25.8
Fall	S11_cal100	17/05/2012	27°01,753	70°50,921	105	13	0.54	−270	−65	15.8
Fall	S14_vlp100	12/05/2012	33°00,921	71°37,333	100	10	1.2	−118	165	12.7
Summer	S3_vlp50	09/01/2012	33°01,485	71°37,325	65	13.5	0.83	−100	80	11.1
Summer	S4_vlp100	10/01/2012	33°00,921	71°37,333	80	14.2	0.97	−100	34	11.6
Summer	S1_ccp50	14/12/2011	36°30,783	73°01,083	50	13.4	1.09	−85	18.4	14
Summer	S2_ccp100	15/12/2011	36°30,866	73°07,750	90	13.8	0.39	−120	10	7.6
Spring	S13_ccp50	29/09/2012	36°30,783	73°01,083	50	9	1.8	−96	8	17.5
Spring	S12_ccp100	29/09/2012	36°30,866	73°07,750	90	10	0.8	−78	37	19.2

Sampling points are as iqq, Iquique; antf, Antofagasta; cal, caldera; vlp, Valparaíso; ccp, Concepción, where the prefix is sample(S)-number and the suffix is depth (50/100). A total of 50 and 100 mean the depth in meters of the samples points. TOM is total organic matter. The redox potential of water and sediment were measured in millivolts (mV). *Date (d/m/y/). **The type of sediment in all samples was fine mud.

Sampling was carried out from the small motorboats “Otilia” (CCP), “Antares” (IQQ), and various fishing boats (ANTF, CAL, and VLP), with a small handy gravity mono-corer. Samples were sub-sampled on board with the upper 5 cm of sediment transferred to sterile tubes and kept at 4°C until DNA extraction (<12 h later). In addition, dissolved oxygen, from overlying water, sediment redox potential (Figures 1B,C), overlying water redox potential (using Ag/AgCl as reference electrode), and temperature, were immediately measured with an Oakton P650 multi-parameter instrument. Porosity and total organic matter (TOM) (Figure 1D) were determined by the ignition method (Mook and Hoskin, 1982).

### DNA extraction and sequencing

DNA extraction was carried out (in triplicate) from 0.5 g of the upper 5 cm of sediments using the Fast DNA Spin Kit for Soil (MP laboratories) and quantified using a Nanodrop ND1000. Next, at the Marine Biological Laboratory (BPC-MBL), Woods Hole, MA, USA, the v4v6 region of the 16S rRNA gene was amplified and sequenced using a 454 GS FLX Titanium sequencing platform, Details of sequencing technology are described in Huber et al. (2007) and Huse et al. (2008). Data and experiments are available under project code PRJNA251688 in the NCBI database. Reads were demultiplexed and barcodes were removed for submission.

### Quality control and filtering reads

After sequencing, the single-end raw reads were assessed for quality control using the FastQC package version 0.11.9,^1^ while the filtering and trimming were performed through the package PRINSEQ-lite version 0.20.4 (Schmieder and Edwards, 2011). Next, reads were subjected to denoising/chimera removal and clustering into amplicon sequence variants (ASVs) using the *denoise-single* method of the DADA2 package, version 1.14 (Callahan et al., 2016), as a plugin through the bioinformatics platform QIIME2, version 2019.10 (Bolyen et al., 2019). The ASV taxonomy assignment was performed through the feature-classifier classify-consensus-vsearch method in QIIME2, using the SILVA database 138.1 as reference (Quast et al., 2012). Singletons were removed from the final ASV table.

### Data analyses

The main data analysis was performed using the R software environment version 3.6.3. Thereby, the bacteria abundance table was rarefied to the smallest sample size (8,000 reads per sample), to perform diversity and ordering analysis, using the function rarefy_even_depth of the Phyloseq package (R package), version 1.30.0 (McMurdie and Holmes, 2013). This function uses the standard R sample function to resample from the abundance values in the ASVs table so that all samples have the same size.

The NMDS ordination analysis was performed using the function ordinate of the package Phyloseq version 1.30.0, using the unifrac and Bray-Curtis distance indices on the ASV abundance table. In particular, unifrac accesses the abundance table, but also to a phylogenetic tree, previously built using the representative reads through QIIME2 and the *de novo* phylogenetic tree creation method, phylogeny fasttree.

The Shannon diversity index and observed ASVs were obtained using the R package Vegan version 2.5-7 (Oksanen et al., 2013). The Analysis of Similarity (ANOSIM) test was used to validate the ordination results using the Vegan function anosim, which statistically tests whether there is a significant difference between two or more groups of sampling units. The statistical comparisons between factors and groups were performed through the Vegan function Adonis statistical test (Permutational Multivariate Analysis of Variance) and the R package ALDEx2 version 1.22.0 (Fernandes et al., 2014). In addition, the correlation and statistical significance between the environmental variables and the Bray-Curtis dissimilarity matrix of the abundance table were performed using a combination of the bioEnv function and the Vegan package’s Mantel test. To evaluate the average percentage contribution of individual bacteria to the dissimilarity between groups in the Bray-Curtis dissimilarity matrices, the Vegan method of similarity percentage decomposition (SIMPER) was performed. The graphical representation of the data was achieved using ggplot2 version 3.3.3 (R package), Circos version 0.69-8 (Krzywinski et al., 2009), library Matplotlib from Python version 3.6, and the R package Metacoder version 0.3.4 (Foster et al., 2017).

## Results

### Community structure and diversity

**FIGURE 2:**
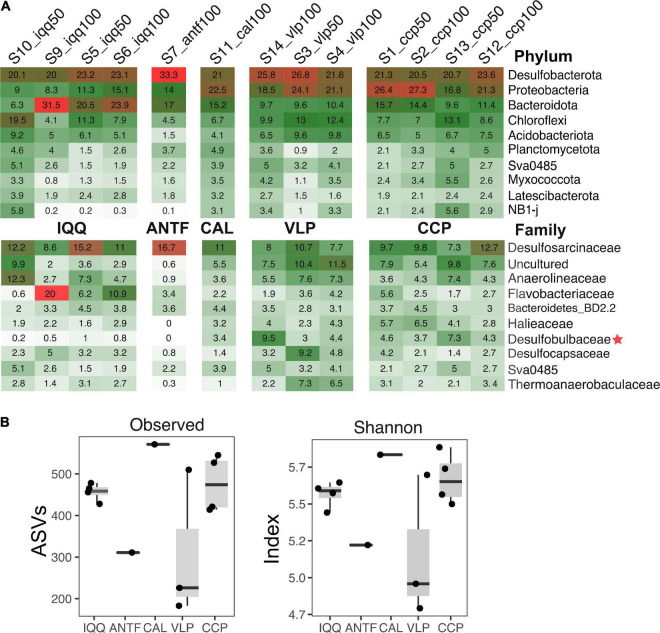
**(A)** Abundance of the ten most abundant phyla and families. The numbers in tiles represent the relative abundance in percent. The red star highlights taxa with statistically significant differences in abundance according to Aldex 2. **(B)** Number of observed ASVs and Shannon diversity index.

The thirteen preprocessed libraries rendered 202,779 reads and 3,614 ASVs. The ASVs were distributed among 55 phyla, where the phylum *Desulfobacterota* (formerly the class *Deltaproteobacteria* within the phylum *Proteobacteria*) dominates with 22.7% of the total abundance, including several sulfur-associated bacteria. Next come the phyla *Proteobacteria* (18.4%), *Bacteroidota* (formerly the class *Bacteroidetes*), (15.8%), and *Chloroflexi* (9.4%) (Figure 2A). In this connection, there were no statistically significant differences between the most abundant phyla (>2% in abundance) and localities (*p*-value > 0.05), evaluated through the ALDEx2 statistical package.

A total of 328 families were identified, where *Desulfosarcinaceae* (11%), in the phylum *Desulfobacterota*, is the most abundant; followed by an uncultured-unlabeled cluster (6.1%), and *Anaerolineaceae* (5.4%), of the phylum *Chloroflexi* (Figure 2A). According to ALDEx2, the family *Desulfobulbaceae* (3.2%) presents statistically significant differences in the abundances between IQQ and VLP-CCP localities (*p*-value < 0.05). On the other hand, although there were not statistically significant differences, the family *Flavobacteriaceae* (5.3%) presented greater abundance in IQQ than in VLP and CCP, while the *Halieaceae* family abundance was greater in VLP and CCP than in IQQ (see Supplementary Figures 2A,B for the ranking of the most abundant classes and orders). It is worth mentioning that although the *Thiotrichaceae* family is not among the ten most abundant families, it had 1.4% of the total abundance, ranking among the 20 most abundant and appearing with an average of 1.9% in CCP. This family harbors some of the most conspicuous large filamentous sulfur bacteria in the area, such as *Ca*. Marithioploca araucae, of the *Gammaproteobacteria* class.

Continuing with the taxonomic assignment, 397 different genera were retrieved (including one cluster for the uncultured and another for the unassigned). The most abundant genus was the Sva0081 sediment group (4.2%), in the family *Desulfosarcinaceae* (Supplementary Figure 2C). In this regard, it should be mentioned that after the Sva0081 sediment group, the genera SEEP-SRB1 (2.25%), LCP-80 (1.19%) and *Desulfosarcina* (0.86%) were identified in the family *Desulfosarcinaceae* (Supplementary Figure 3). While in the family *Anaerolineaceae* (5.4%), six genera were identified, of which *Pelolinea* (3.1%) was the most abundant and cosmopolitan. Furthermore, IQQ presented the largest diversity and abundance within that family (Supplementary Figure 3). Moreover, going down to the ASV ranking, the most frequent ASV was assigned to the genus *Lutimonas* (1.5%) in the phylum *Bacteroidota*, and the second in the frequency ranking (1.4%), to the order *Syntrophobacterales*, phylum *Desulfobacterota* (the graphical distribution of the most frequent thousand ASVs is in Supplementary Figure 1).

Compared with the rest of the sampling stations ANTF presented some different taxa with opposite abundances. Thus, the maximum abundance of *Desulfobacterota* and its family *Desulfosarcinaceae* was found in ANTF, while the abundance of families such as *Anaerolineaceae* (5.4%) was very low (0.9%), and others, such as *Halieaceae* and *Desulfobulbaceae* (which were present in IQQ, CAL, VLP, and CCP), were absent.

Regarding diversity indexes (Figure 2B), no statistically significant differences were found between the sampling stations (*p*-value > 0.05), although VLP had the lowest number of ASVs and Shannon diversity index, in which the sample s14_vlp100 was notably higher than s3_vlp100 and s4_vlp50, increasing the variance in the VLP locality. Furthermore, in terms of numbers of ASVs and Shannon diversity index, the localities of IQQ, CAL, and CCP, were very similar.

### Spatial pattern

**FIGURE 3:**
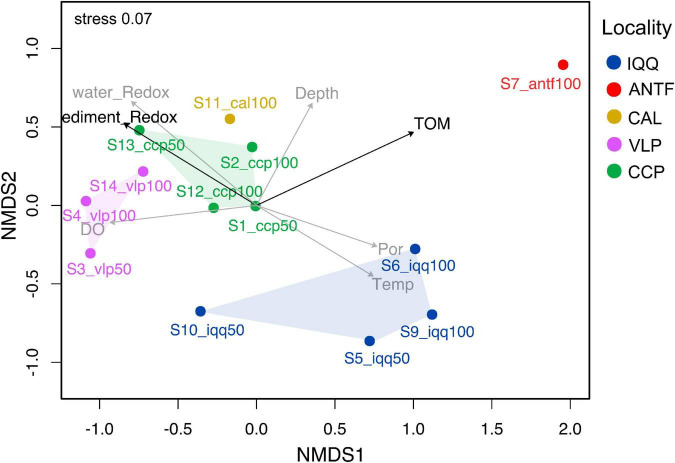
Non-metric multidimensional scaling (NMDS) plot of the Bray-Curtis distance of the bacterial community with environmental parameters added using envfit. The NMDS was constructed over the Bray-Curtis dissimilarity matrix. The data was rarefied at 8,000 sequences per sample. The black arrows represent a statistically significant relationship (*p* < 0.05) measured through the Mantel or envfit test. The added parameters were the redox potential of sediment and water, the percentage of total organic matter (TOM), depth, temperature (Temp), and porosity (Por).

The NMDS analysis of the unifrac and Bray-Curtis distance matrix revealed a spatial pattern, in which the sampling points of IQQ, VLP, and CCP generated separate clusters (Figure 3). Although only localities with replicate samples (IQQ, VLP, and CCP) were considered valid for *post-hoc* analysis, it should be noted that the ANTF sampling point appeared distant from the complementary sampling points, while CAL appeared close to CCP. The *post-hoc* ANOSIM test confirmed that the clusters observed in the NMDS were statistically significant (*R* = 0.56, *p*-value = 0.0017), validating the spatial pattern.

Regarding the influence of environmental measures on the Bray-Curtis dissimilarity of the microbial community, the percentage of TOM appeared as the most relevant variable (Supplementary Table 1), as is shown in the adjusted NMDS ordination analysis, built through the envfit method and the metaNMDS function from R (Figure 3). Furthermore, the bioEnv and Mantel test confirmed that TOM has a statistically significant positive correlation with bacterial abundance (*r* = 0.36, *p*-value = 0.02) as well as redox potential (*r* = 0.27, *p*-value; *p* = 0.02) (Supplementary Table 2).

To assess how taxa and, in particular, how families influenced the spatial pattern, the ten most abundant families (48% accumulated abundance) were evaluated through the Adonis statistical test, including the IQQ, VLP, and CCP localities as factors. The result showed that the family *Desulfobulbaceae* was the most important explanatory variable (Supplementary Table 3) for spatial pattern between the localities (*R*^2^ = 0.61; *p*-value < 0.01). Moreover, the second most important explanatory variable was *Bacteroidetes* BD2-2 (*R*^2^ = 0.58; *p*-value < 0.01), followed by *Thermoanaerobaculaceae* (*R*^2^ = 0.57; *p*-value < 0.01), and *Desulfocapsaceae* (*R*^2^ = 0.55; *p*-value < 0.01). Besides, at the ASV level, the SIMPER analysis showed that the greatest contribution to the dissimilarity pattern between IQQ and CCP was generated by a couple of ASVs assigned to the genus *Lutimonas*, one ASV assigned to *Syntrophobacterales*, one to the genera *Desulfonema* and *Sulfurovum*, which made 13.6% of the total contribution to dissimilarity. Differentially, between IQQ-VLP and CCP-VLP the greatest contribution was made by an ASV assigned to the genera B2M28 (class *Gammaproteobacteria*) (Supplementary Table 4).

### The family *Desulfobulbaceae*

Since the family *Desulfobulbaceae* appeared as the most important taxonomic explanatory variable for the spatial pattern between localities, we investigated the distribution of the representatives of this family identified throughout the localities under study.

**FIGURE 4:**
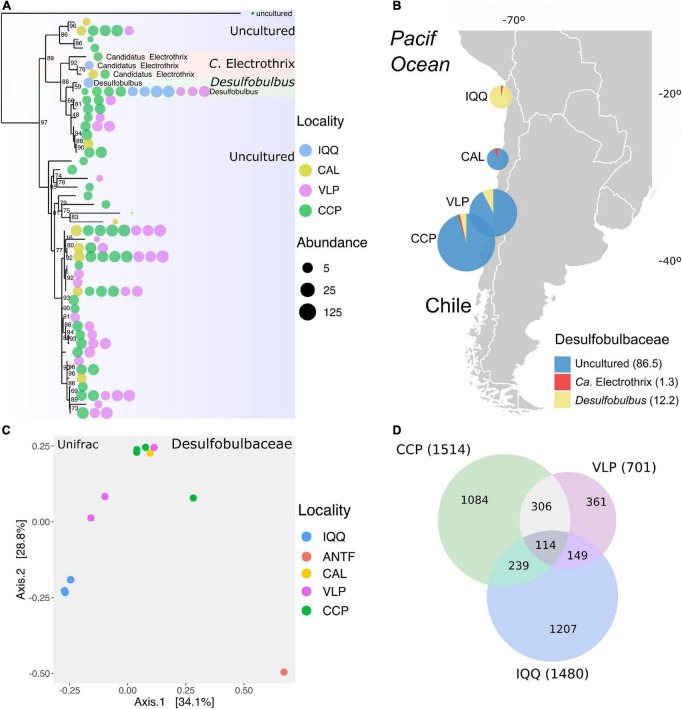
**(A)** Phylogenetic tree of the representatives of the family *Desulfobulbaceae* identified across the data. The color and size of the bubbles indicate the presence and abundance of taxa by locality. **(B)** Distribution of genera of the family *Desulfobulbaceae* by locality in percentage **(C)** Non-metric multidimensional scaling (NMDS) based on the unifrac matrix distance of the abundance in the *Desulfobulbaceae* family (Stress = 0.0005). **(D)** Venn chart of shared and unique ASVs across all data by locality. The number in parentheses is the total ASVs.

With 3.2% of the total abundance and 47 ASVs, the family *Desulfobulbaceae* contained three ASVs assigned to the genus *Ca*. Electrothrix, in the cable bacteria functional group, and two others, to the genus *Desulfobulbus*; the complement of 43 ASVs are uncharacterized forms (uncultured). In this regard, the phylogenetic relationship based on the ASV sequences showed four uncultured ASVs in the *Ca*. Electrothrix clade, seven in the *Desulfobulbus* clade, and the rest in a separate clade (Figure 4A). The *Ca*. Electrothrix-assigned ASVs were present in four samples from IQQ, CAL, and CCP, while *Desulfobulbus* was present in eleven samples from IQQ, VLP, and CCP, thus more ubiquitous and abundant than *Ca*. Electrothrix (Figure 4B). The uncultured genus ASVs were present in the CAL, VLP, and CCP sampling sites, and remarkably, IQQ was characterized by the almost exclusive presence of *Desulfobulbus*. Thus, the distribution pattern of *Desulfobulbaceae* emphasizes the general spatial pattern while further separating the IQQ cluster (Figure 4C). Sampling locality differences become more obvious when the numbers of shared and unique ASVs are considered, since only one *Desulfobulbus* assigned ASV (2.1%), is shared among IQQ, VLP, and CCP (Supplementary Figure 4). Concerning the influence of environmental variables on the abundance pattern of *Desulfobulbaceae*, the redox potential resulted in a statistically significant positive correlation (*r* = 0.41; *p*-value = 0.001).

### Shared and unique amplicon sequence variants

The latitudinal spatial pattern observed at the ASV level in the *Desulfobulbaceae* family, especially in IQQ, led to evaluating the shared and unique ASVs among IQQ, VLP, and CCP across the whole data. Removing the ANT and CAL data the ASVs shared among IQQ, VLP, and CCP were only 113 out of the total of 3,017 ASVs, which represent only 3.7% of the total ASVs (Figure 4D), and 26% of the total reads. Taxonomically, the phylum *Desulfobacterota* (23.5%) dominated among shared ASVs, followed by *Bacteroidota* (19.4%) and *Proteobacteria* (16.2%). Moreover, at the individual level, the first and third most abundant shared ASVs were assigned to the genus *Lutimonas* of the phylum Bacteroidota, and the second, to the phylum *Desulfobacterota* (order *Syntrophobacterales*).

**FIGURE 5:**
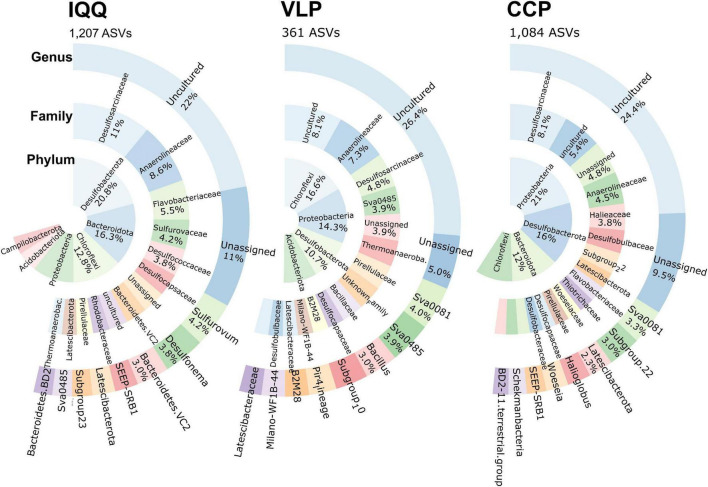
Relative abundance of the most frequent unique ASVs and its taxonomic assignment at the phyla, family, and genus levels by locality.

From the uniqueness perspective, IQQ had the highest proportion of unique ASVs (Figure 5) (40%), closely followed by CCP with 36% and, both distant from VLP with 12%. Taxonomically, the phylum *Desulfobacterota* (20.8%) contained the larger number of unique ASVs in IQQ, while in CCP was *Proteobacteria* (21%), and in VLP *Chloroflexi* (16.6%).

At the family level, and without considering the uncultured and the unassigned, the *Desulfosarcinaceae* and *Anaerolineaceae* contained the larger number of unique ASVs. After them, the family *Flavobacteriaceae* (5.5%) in IQQ; *Halieaceae* (3.8%) in CCP, and Sva0485 (formerly *Deltaproteobacteria Candidate* Sva0485 clade) in VLP appeared as the most abundant.

At the genus level and on the basis of unique ASVs, samples from IQQ, VLP, and CCP have a prominent sulfidic profile, with the presence of *Sulfurovum* (4.2%) (SOB) and *Desulfonema* (3.8%) (SRB) at the top of the abundance in IQQ, while, VLP and CCP coincide with the genus Sva0081 sediment group (SRB) as top taxa (after the unassigned and uncultured) (Figure 5). Conversely, in IQQ the genus Sva008 sediment group presented low abundance. In this sense, uncultured and unassigned assignments, ranging 31–35% of the accumulated abundance, leaves 65–69% of the sequences with known taxonomic affiliation at the genus level.

## Discussion

### Community structure and diversity

This study shows that the best represented bacteria phyla were *Desulfobacterota* (23%) (formerly the class *Deltaproteobacteria*), *Proteobacteria* (18%), *Bacteroidota* (16%) (the former phylum *Bacteroidetes*), and *Chloroflexi* (9%), a distribution that differs with descriptions of similar environments, such as the superficial anoxic OMZ sediments of the Arabian Sea and the Bay of Bengal (Lincy and Manohar, 2020), where *Firmicutes* (33.08%), *Proteobacteria* (32.59%), *Bacteroidetes* (17.48%), and *Chloroflexi* (5.52%) were the most abundant phyla. The *Firmicutes* phylum appears with only 1.5% of the total abundance in the present study, which may be attributable to patchy distribution or, as Cupit et al. (2019) posits, the fact that a wide range of *Firmicutes* species are not captured by conventional culture-independent DNA extraction methods, primarily due to the non-germinative state of *Firmicutes* endospores.

Global studies on the deeper anoxic layers, i.e., the subseafloor (Hoshino et al., 2020) have reported *Chloroflexi*, *Planctomycetes*, and *Ca*. Atribacteria (*Ca*. Caldatribacteriota or OP9/JS1) as the most frequent phyla. Indeed, in anoxic subseafloor sediments, *Ca*. Atribacteria is described as one of the most abundant bacterial taxa (Orsi, 2018; Hoshino et al., 2020), although it was scarcely present in the HS surface sediment samples. The most likely reason is that *Ca*. Atribacteria were distributed deeper in the sediments, whereas this study covers the first 5 cm of sediment.

In the HS the dominant classes were similar to those reported by Zinger et al. (2011) in their study of coastal sediments, where the most abundant classes were *Gammaproteobacteria* and *Deltaproteobacteria*, which are equivalent to the most abundant classes identified in the HS, taking into account that *Desulfobacteria* is the former *Deltaproteobacteria* class. Therefore, the HS community presents distinctive features at the phylum level with respect to similar environments, while at the class level it seems to agree with previous reports.

The two most abundant families in the HS, the *Desulfosarcinaceae* (10.9%) and *Anaerolineaceae* (5.4%) appear to thrive relatively well in this primeval sulfidic (anoxic) type of environment, a factor that supports the micropaleontological claim relating to the findings of filamentous microfossils in the Hadean (3,235 and 3,465 Ma ago) (Rasmussen, 2000; Schopf et al., 2018). The novel family *Desulfosarcinaceae* includes genera such as *Desulfosarcina*, *Desulfatitalea*, SEEP-SRB1, and Sva0081 sediment group, all of them sulfate-reducing bacteria (SRB). Further, the Sva0081 sediment group was the HS’s most abundant genus. According to previous studies, this uncultured genus is an important sink for acetate (Dyksma et al., 2018a) and for H_2_ in coastal marine sediments (Dyksma et al., 2018b). It proliferates fast in microaerobic conditions using the H_2_ that diffuses from fermentation deeper in the sediment (Dyksma et al., 2018a). Therefore, the dominant presence of Sva0081 sediment group in the HS suggests that H_2_ consumption would be an important metabolic pathway for energy, supporting populations like the Sva0081 sediment group.

As mentioned above, in the HS, the family *Anaerolineaceae* (5.4%) in the phylum *Chloroflexi*, was the second most abundant family after the *Desulfosarcinaceae*, with six genera. Among these, *Pelolinea* (3.1%), an anaerobic, cosmopolitan, filamentous genus (Imachi et al., 2014), was the most common. Most of the *Anaerolineaceae* reads (90.3%) are unknown and are assigned as uncultured bacteria. In general terms, this family is defined as consisting of strictly anaerobic and chemoheterotrophic multicellular filamentous bacteria, mostly using sugars and protein for fermentation (Yamada and Sekiguchi, 2018). In this connection, *Anaerolineaceae* could be playing an important role in the HS by generating organic acids, such as acetate (Liang et al., 2015). The latter molecule has been indicated as one of the most important energy substrates for sulfate-reducing bacteria in marine sediments (e.g., Parkes et al., 1989).

At the ASV level, the most frequent in the HS was assigned to the genus *Lutimonas*, which is an interesting coincidence with the Black Sea sediment bacterial community where it has been reported as the most abundant OTU (Jessen et al., 2017). Moreover, the genus *Lutimonas* is defined as symbiont, first isolated from the polychete *Periserrula leucophryna* (Yang et al., 2007), with variable capacity for nitrate reduction (Kim et al., 2014), and presumably also, nitrogen fixation (Petersen et al., 2016). Although the dominant presence of the symbiont genus *Lutimonas* in different sediments has been reported (Quero et al., 2015), its high abundance in the HS suggests that this local taxon is a free-living bacterium, not yet characterized.

This study further shows that the diversity and species richness in both the IQQ and CCP sampling stations are similar, while the VLP station presented the lowest diversity and relative abundances. This feature might be explained by the narrow shelf and thus of the sublittoral zone at this point, as compared with that off Concepcion and off Valparaiso (Völker et al., 2012) which in turn may be related to the quality and levels of available TOM. As suggested by Orsi (2018), these variables generally correlate with the number of cells and diversity in the sediments which depends on the lower or greater availability of electron donors in higher organic carbon fluxes.

### Geographic patterns

The NMDS and ANOSIM analyses showed a spatial pattern that separated the sampling localities. Thereby, IQQ and CCP on one side and VLP on the other. In this regard, TOM and redox potential were statistically significantly correlated with bacterial abundance, in this case favoring the former two locations which shared productivity-enhancing factors, i.e., primary productivity-enhancing upwelling in IQQ. In addition to this factor, the nutrient input from the regional rivers (most important, the Bio-Bio and Itata rivers) may play a key factor at Concepcion. It is argued that productivity regimes, especially the content of organic matter in sediments, as well as the presence of oxygen, are key in shaping the structure and diversity of bacterial communities (Bienhold et al., 2016; Hoshino et al., 2020), changing spatially, even at microscales as a result of aggregation, creating very complex communities (Zorz et al., 2019).

Regarding spatial distribution patterns and taxa, the family *Desulfobulbaceae* turned out to be the most influential taxon in explaining the spatial pattern of the HS bacterial community, while it is the seventh most abundant family (3.2%). Indeed, this family presented statistically significant differences in abundance, where its presence is scarce in the locality of IQQ compared to VLP and CCP. In this regard, it has been shown that, as expected, the family *Desulfobulbaceae* tend to enhance their relative abundance toward anoxic conditions, especially those groups affiliated with sulfate-reducing bacteria, since the reduction of sulfates dominated the remineralization of organic matter with increasing hypoxia (Thamdrup and Canfield, 1996; Jessen et al., 2017). It is noteworthy mentioned that the family *Thermoanaerobaculaceae*, containing thermophilic bacteria, is present in all the sampling points of this study, and is the third most influential taxon in explaining the spatial pattern. In this regard, three uncultured genera were identified across the data; Subgroup 10, Subgroup 23 and TPD-58. According to Dedysh and Yilmaz (2020), sequences that share similarities with the 16S rRNA gene sequence of *Thermoanaerobaculum aquaticum* MP-01^T^, the only described member of the family, have been recovered from a variety of habitats including hot springs, saturated soils of water and sediment. The family *Thermoanaerobaculaceae* appears as ubiquitous in all localities and represent an interesting future focus of study in this environment.

### The family *Desulfobulbaceae*

The family *Desulfobulbaceae*, in the phylum *Desulfobacterota*, includes SRBs and SOBs, and it has recently been suggested that it maintains important H_2_ scavengers subgroups (Dyksma et al., 2018b). Members of this family are commonly found in marine sediments at different latitudes, i.e., the Baltic Sea (Marzocchi et al., 2014; Marshall et al., 2019; Dam et al., 2021), and off the coast of Chile, e.g., in the coastal ecosystem off the Atacama desert, where between some points the *Desulfobulbaceae* is one of the dominant and key families (Zárate et al., 2021).

The data corresponding to *Desulfobulbaceae* contains ASVs assigned to the genera *Desulfobulbus*, *Ca*. Electrothrix (Trojan et al., 2016), and uncultured forms. The genus *Desulfobulbus* is strictly anaerobic, having both a respiratory and fermentative metabolism, with several ways to get energy, i.e., chemoorganoheterotrophic or chemolithoheterotrophic growth. Moreover, sulfate, sulfite, and often thiosulfate serve as terminal electron acceptors and are reduced to H_2_S (Galushko and Kuever, 2019). On the other hand, the genus *Ca*. Electrothrix corresponds to filamentous SOB, within the cable bacteria functional group, capable of conducting electrons over centimeter-long distances, coupling sediment electrogenic sulfur oxidation (e-SOx) to dissolved oxygen (Nielsen et al., 2010; Pfeffer et al., 2012), nitrate and nitrite reductions (Marzocchi et al., 2014). Cable bacteria have been found in different oceanographic settings and climate zones, and, in several different coastal habitats (Burdorf et al., 2017), and are a topic of increasing interest. Its presence in the HS system suggests that e-SOx could be occurring in this system, mostly sustained by high levels of nitrate (>20 μM) in both sediments Ahumada et al., 1983), and water column (see also Annexes in Gallardo et al., 2013b).

The relative abundance of *Desulfobulbaceae* in IQQ is low compared to those in VLP and CCP localities. Since *Desulfobulbus* represents 95% of the abundance of *Desulfobulbaceae*, along with a small abundance of *Ca*. Electrothrix (1 ASV; 5% of the abundance) in IQQ. Furthermore, unknown ASVs, assigned as uncultured *Desulfobulbaceae*, were not present in IQQ. Beyond that fact, the uncultured *Desulfobulbaceae* groups represent a “black box,” probably new species, close to *Desulfobulbus* and *Ca*. Electrothrix (Figure 4A).

The spatial pattern that separates IQQ, VLP, and CCP is clear in the family *Desulfobulbaceae*, where the redox potential appeared positively correlated. In fact, the redox potential is more negative in the northernmost locations (IQQ and ANTF) than in the Central-Southern locations. Therefore, the representatives of the *Desulfobulbaceae* family thrived better in more oxidized sediments in the HS.

The degree of shared *Desulfobulbaceae* ASVs was very low, thus only one ASV (*Desulfobulbus*) is shared between IQQ, VLP, and CCP localities, representing a large latitudinal fragmentation of the group among sites.

### Sharing and uniqueness in the community

The spatial fragmentation of the community is clearer at the finer grain phylogenetic level as represented by the ASV clusters, which theoretically span species and subspecies layers. Therefore, the ANOSIM value for the spatial pattern is maximum at the ASV level (Supplementary Figure 5), supporting the idea that the spatial pattern is a better fit at this deep phylogenetic level.

This fine spatial pattern, at the ASV level, is probably an extended and general rule for sediment microbial communities, better reflecting environmental changes in microscales. In the Baltic Sea, for example, each site contained distinct ASV signatures of unique sulfate-reducing microorganisms in each family (Marshall et al., 2019). Along the same lines, the percentage of shared ASVs among IQQ, VLP, and CCP is very low, i.e., 3.7% (26% of the reads). This level is even lower than the shared ASVs (16.8%), between anaerobic and aerobic bacterial communities in subseafloor sediments (Hoshino et al., 2020), and those for the Bay of Bengal OMZ and Arabian Sea OMZ sediment bacterial community, with 30% shared OTUs (Lincy and Manohar, 2020). Thus, this result suggests the HS contains a high level of microdiversity, consistent with the Thienemann-Sander temporal stability hypothesis (Gallardo et al., 2016), but with microscale spatial changes, which drive the high microdiversity in a general context of stability, characterized by high abundance of sulfur species, nitrate and very low or no oxygen concentration.

The unique-shared perspective allows distinguishing those ASVs under the same taxonomic assignment, but present and absent in specific locations, revealing intraspecies variation, reflecting high microdiversity across the community. In this regard, IQQ was the locality with the highest proportion of unique ASVs (40%) and, although in a different order, they appeared predominantly assigned to *Desulfobacterota*, *Proteobacteria*, and *Chloroflexi*, and further, the most frequent families were *Desulfosarcinaceae*, and *Anaerolineaceae* (in the phylum *Chloroflexi*). Thus, at each site, there is a strong selection for taxonomically lower-level bacteria into the same groups (micro niches). Moreover, at the genus level, in the IQQ site, the most abundant unique ASVs were assigned to the genera *Sulfurovum* and *Desulfonema*, a facultative anaerobic SOB (hydrogen-oxidizing, thiosulfate-reducing), and SRB, respectively. In the VLP sampling site, the anaerobic SRB genera Sva0081 sediment group and Sva0485 (*Candidatus* Acidulodesulfobacterales), a facultatively anaerobic, sulfate/iron reducer, probably a sulfur oxidant under aerobic respiration (Tan et al., 2019), dominated the unique ASVs. In the CCP site, the Sva0081 sediment group was the most frequent, while poorly represented in IQQ suggesting different biogeochemical processes than those at VLP and CCP. Thus, according to these observations, both sulfur-reducing and sulfide-oxidzing processes occur simultaneously.

## Conclusion

According to this study *Desulfobacterota* and *Desulfosarcinaceae* dominate in the HS, especially by SRB, reflected by the most abundant genus: the uncultivated genus Sva0081 sediment group. The revealed spatial pattern along the Chilean coast indicates changes at the bacteria community level, positively correlated with the percentage of TOM and redox potential.

Despite the fact that the *Desulfobulbaceae* species are still not properly characterized, they appeared as a key taxon with significant differences in their distribution influencing the general spatial pattern. Moreover, the spatial pattern is influenced by a large microdiversity, since a very small number of ASVs are shared (3.4%) among the localities included in the comparison, reflecting a diverse, mature and stable HS bacterial community.

## Data availability statement

The datasets presented in this study can be found in online repositories. The names of the repository/repositories and accession number(s) can be found below: https://www.ncbi.nlm.nih.gov/bioproject/PRJNA251688, PRJNA251688.

## Author contributions

AF, VAG, and IPGM: conceptualization. AF: formal analysis. VAG, CE, and LN: resources. LPN, IPGM, and VAG: funding acquisition and supervision. AF, CE, and VAG: experimental studies and writing—original draft. AF, VAG, IPGM, and LPN: wrote and finalized the manuscript. All authors have read and approved the final version of the manuscript.

## Footnotes

1
https://www.bioinformatics.babraham.ac.uk/projects/fastqc/
