# Supplementary material for: Bacterial community of sediments under the Eastern Boundary Current System shows high microdiversity and a latitudinal spatial pattern
Source: Front Microbiol. 2022 Sep 30;13:1016418. doi: 10.3389/fmicb.2022.1016418 (PMC9561620; doi:10.3389/fmicb.2022.1016418)
Supplement: Supplementary file 1 [file Data_Sheet_1.PDF]

Supplementary Material

1 Supplementary Figures and Tables

1.1 Supplementary Figures

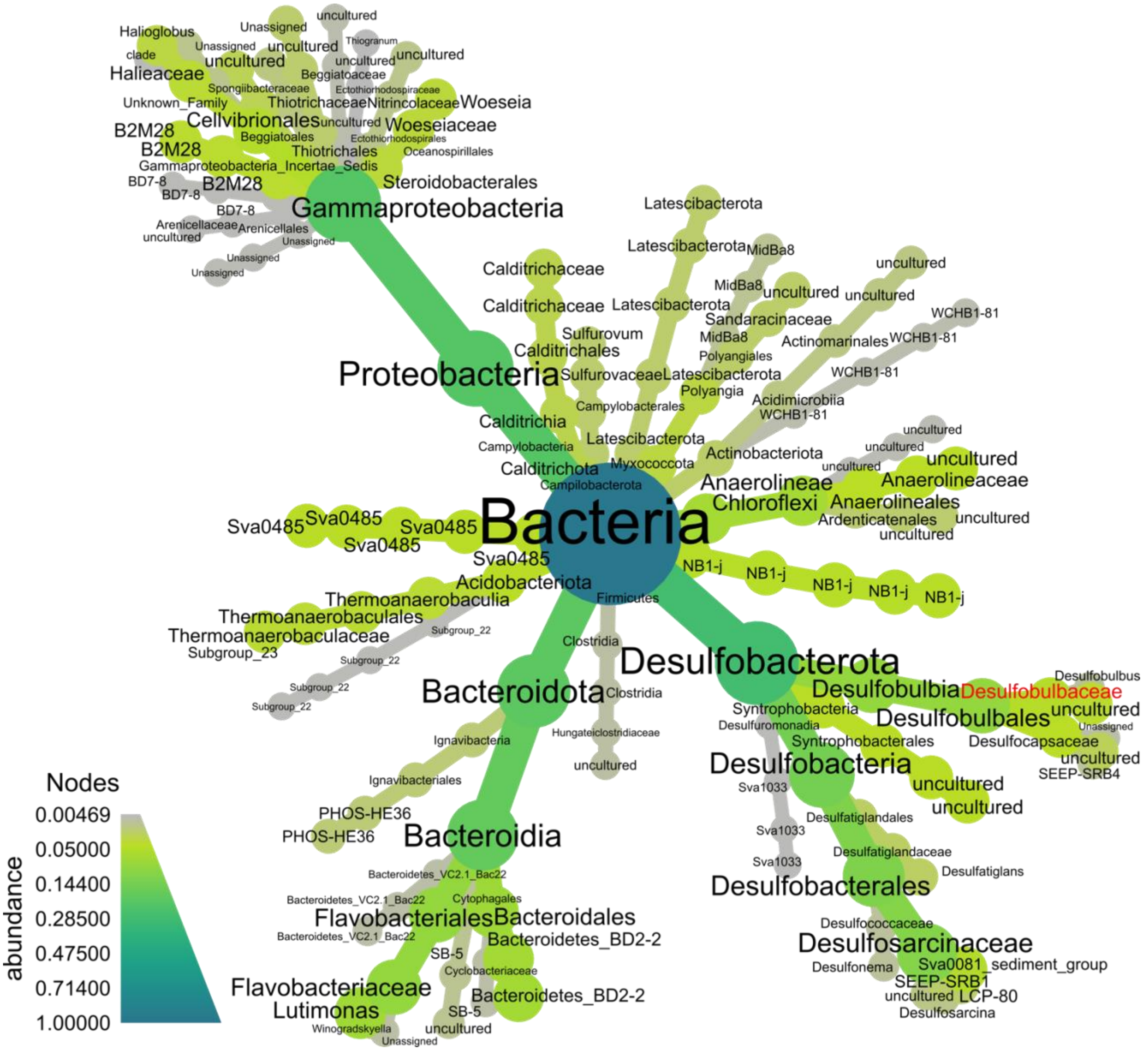

Supplementary Figure 1. Distribution of the thousand most abundant ASVs through the data. The color and size of the nodes and internodes represent the abundance, being maximum in the central node (Bacteria). The central node is the Domain, then is phylum, Class, Order, Family, and genus.

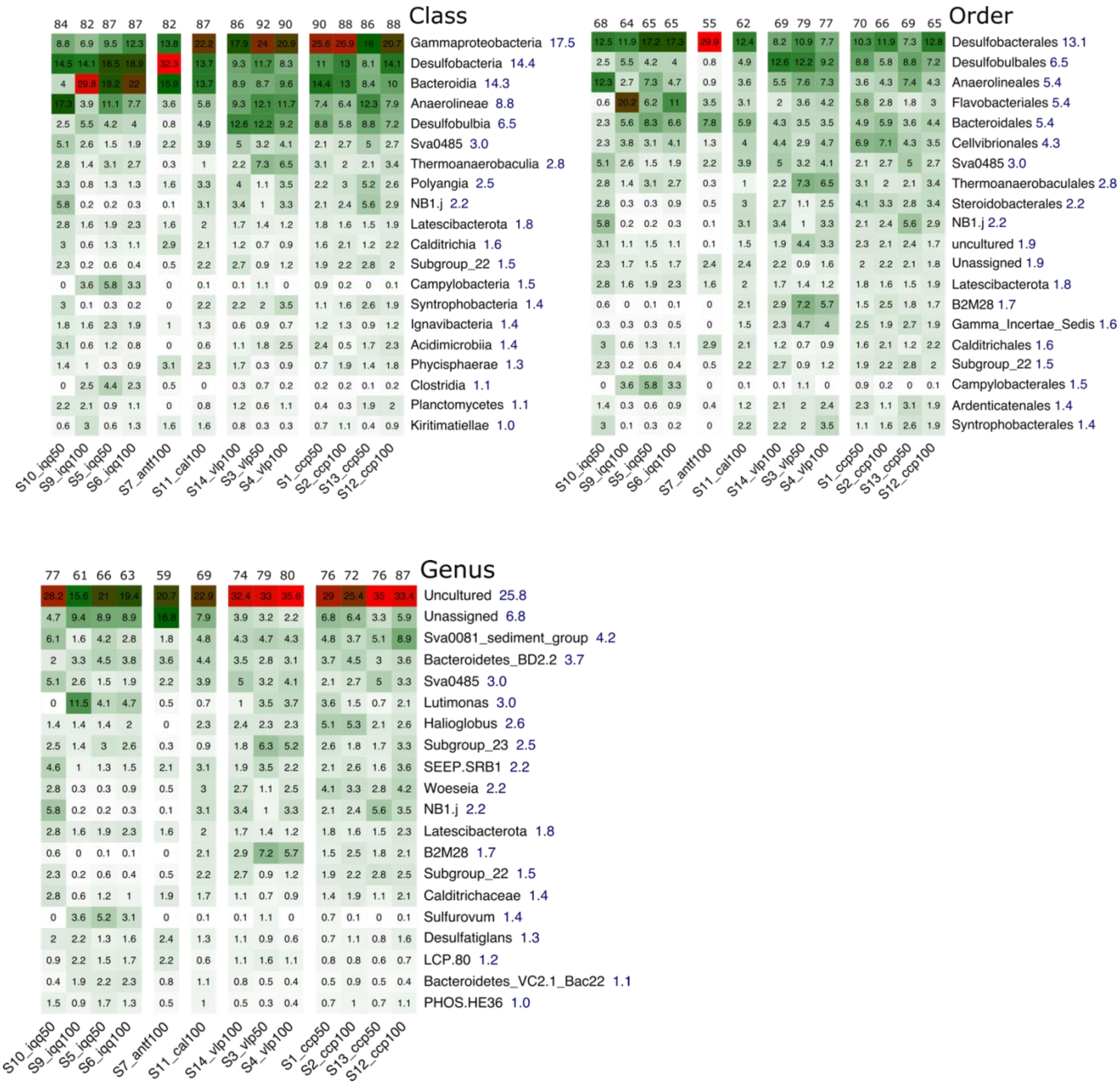

Supplementary Figure 2. Ranking of the 20 most abundant classes, orders, and genera throughout the data. The blue numbers are the percentage abundance in the general context and the tiled numbers are the relative abundance in the samples.

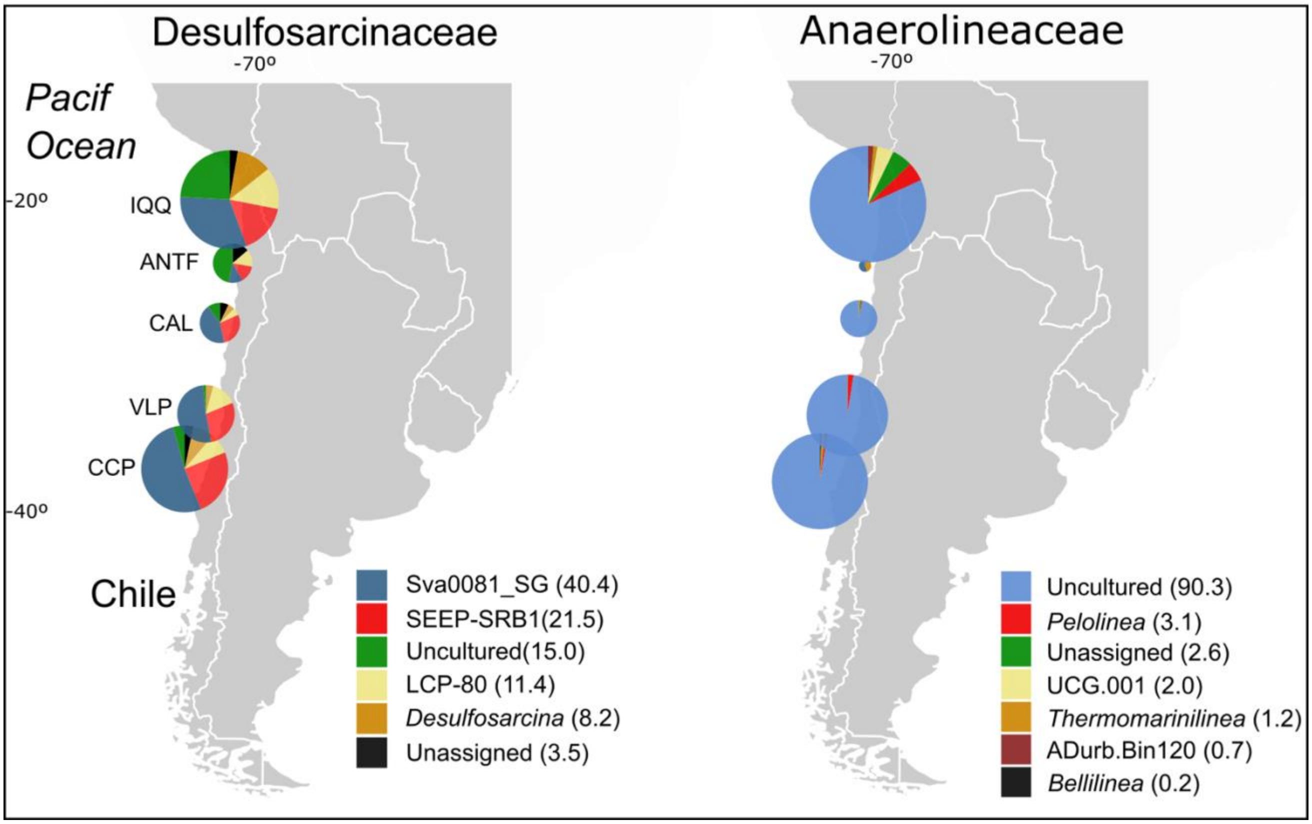

Supplementary Figure 3. Abundance and distribution of the representatives of the families *Desulfosarcinaceae* and Anaerolinaceae through the sampling points. The numbers in parentheses are the percentage abundance in the family.

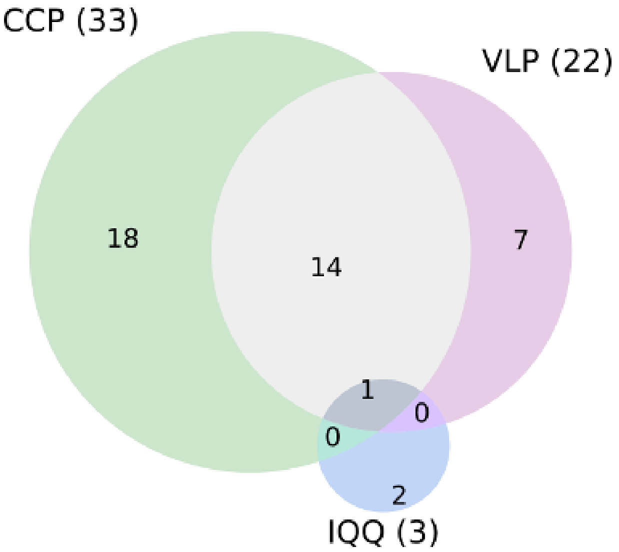

Supplementary Figure 4. Venn plot of shared and unique ASVs in the family Desulfobulbaceae by locality. The number in parentheses is the total ASVs.

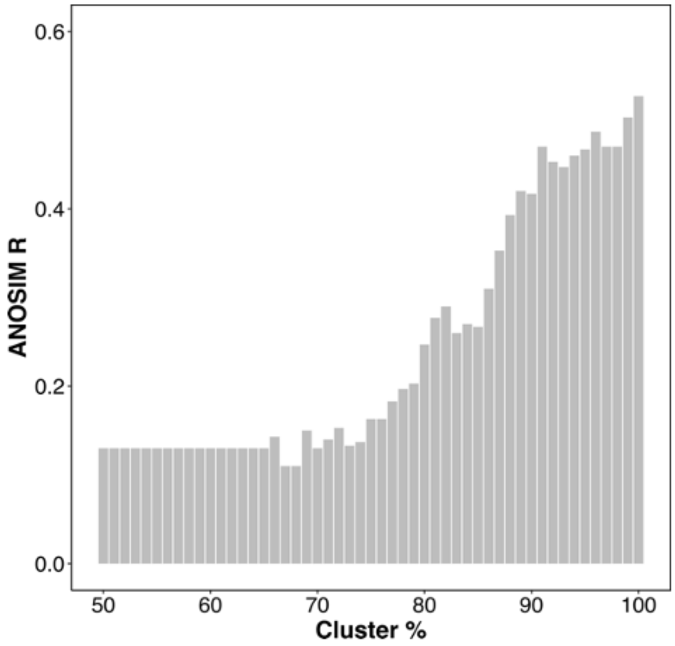

Supplementary Figure 5. ANOSIM value at different levels of the community. Where 100% represents the ASV level.

1.2 Supplementary Tables

Table supplementary 1. Values to the Envfit function (R package), where the correlations are the square root of column r2 and Pr the statistical significance. DO = Dissolved Oxygen and TOM = Total Organic Matter %.

Vectors	NMDS1	NMDS2	r2	Pr(>r)
Depth	0.47882	0.87791	0.2242	0.28429
Temp	0.85528	-0.51816	0.3082	0.16803
DO	-0.9932	-0.11643	0.3602	0.1099
Sediment Redox P.	-0.85105	0.52508	0.3769	0.08538
Water Redox P.	-0.76914	0.63908	0.438	0.06267
TOM	0.90622	0.4228	0.4976	0.02816*
Porosity	0.94676	-0.32195	0.2675	0.21163

*Pr value <0.05.

Table supplementary 2. Correlations between the abundance of the bacterial community and environmental variables, using the Mantel test, with the R and P-value.

Vectors	R	P-value
DO	0.1426	0.19
Sediment Redox	0.2705	0.017*
TOM	0.3857	0.025*
Porosity	0.1824	0.183

P-value <0.05.

Table supplementary 3. Adonis test for the ten most abundant families, with IQQ, VLP, and CCP samples as factors. The R-value represents the proportion in the variance between groups. Pval is the confidence value.

Taxa	ASVs	Mean_relabu	MSLocality	MSres	F.Model	R	Pval
Anaerolineaceae	169	37.37278	0.5384644	0.22319	2.412583	0.5467507	0.002**
Bacteroidetes_BD2-2	62	64.56452	0.3543199	0.1245561	2.84466	0.5871743	0.007**
**Desulfobulbaceae**	**46**	**83.5**	**0.6621927**	**0.1563386**	**4.235632**	**0.6136553**	**0.004****
Desulfocapsaceae	43	88.46512	0.5138506	0.2059511	2.495012	0.5550624	0.001**
Desulfosarcinaceae	142	85.72535	0.4973136	0.2365953	2.101959	0.5124280	0.002**
Flavobacteriaceae	75	78.09333	0.3758931	0.2550084	1.474042	0.4243017	0.094
Halieaceae	44	81.29545	0.4976742	0.2365993	2.103447	0.4409637	0.031*
Sva0485	46	78.97826	0.4727621	0.1986848	2.379458	0.5433224	0.005**
Thermoanaerobaculaceae	47	71.40426	0.4184875	0.1584908	2.640454	0.5690076	0.002**
Uncultured	163	46.22086	0.4482078	0.2419187	1.85272	0.4808863	0.011*

Significant P values: 0 ‘***’ 0.001 ‘**’ 0.01 ‘*’ 0.05 ‘.’ 0.1 ‘ ’ 1

Table supplementary 4. SIMPER analysis showing the 6 ASVs that made the greatest contribution to the dissimilarity between IQQ, VLP and CCP.

IQQ vs CCP

ASV	Taxa	average	sd	ratio	cumsum	P
ASV1	Bacteroidota.c__Bacteroidia.o__Flavobacteriales.f__Flavobacteriaceae.g_Lutimonas	0.99	0.61	1.63	1.17	0.10
ASV4	Bacteroidota.c__Bacteroidia.o__Flavobacteriales.f__Flavobacteriaceae.g_Lutimonas	0.86	0.56	1.55	2.19	0.09
ASV2	Desulfobacterota.c__Syntrophobacteria.o__Syntrophobacterales.f__uncultured.g_uncultured.s__uncultured_bacterium	0.58	0.31	1.88	2.87	0.20
ASV30	Desulfobacterota.c__Desulfobacteria.o__Desulfobacterales.f__Desulfococcaceae.g_Desulfonema.s__uncultured_organism	0.47	0.75	0.63	3.43	0.40
ASV23	Campilobacterota.c__Campylobacteria.o__Campylobacterales.f__Sulfurovaceae.g_Sulfurovum.s__uncultured_bacterium	0.45	0.29	1.53	3.96	0.08

IQQ vs VLP

ASV	Taxa	average	sd	ratio	cumsum	P
ASV6	Proteobacteria.c__Gammaproteobacteria.o__B2M28.f__B2M28.g_B2M28.s__uncultured_bacterium	1.27	0.54	2.36	1.46	0.00
ASV1	Bacteroidota.c__Bacteroidia.o__Flavobacteriales.f__Flavobacteriaceae.g_Lutimonas	1.17	0.46	2.55	2.80	0.03
ASV4	Bacteroidota.c__Bacteroidia.o__Flavobacteriales.f__Flavobacteriaceae.g_Lutimonas	1.06	0.61	1.73	4.02	0.02
ASV2	Desulfobacterota.c__Syntrophobacteria.o__Syntrophobacterales.f__uncultured.g_uncultured.s__uncultured_bacterium	0.71	0.42	1.69	4.83	0.04
ASV8	Sva0485.c__Sva0485.o__Sva0485.f__Sva0485.g_Sva0485.s__uncultured_ bacterium	0.65	0.22	2.92	5.57	0.00

CCP vs VLP

ASV	Taxa	average	sd	ratio	cumsum	P
ASV6	Proteobacteria.c__Gammaproteobacteria.o__B2M28.f__B2M28.g_B2M28.s__uncultured_bacterium	0.97	0.56	1.73	0.01	0.04
ASV20	Proteobacteria.c__Gammaproteobacteria.o__B2M28.f__B2M28.g_B2M28	0.45	0.45	1.00	0.02	0.15
ASV37	Desulfobacterota.c__Desulfobulbia.o__Desulfobulbales.f__Desulfocapsaceae.g_SEEP.SRB4	0.42	0.14	2.93	0.03	0.00
ASV1	Bacteroidota.c__Bacteroidia.o__Flavobacteriales.f__Flavobacteriaceae.g_Lutimonas	0.41	0.26	1.62	0.03	1.00
ASV3	Acidobacteriota.c__Thermoanaerobaculia.o__Thermoanaerobaculales.f__Thermoanaerobaculaceae.g_Subgroup_23	0.37	0.29	1.27	0.04	0.54
